# The association of serum uric acid with severity and prognosis of patients with diabetic foot ulcers

**DOI:** 10.3389/fendo.2026.1732361

**Published:** 2026-04-13

**Authors:** Shu-Min Wang, Yang He, Lin Lin, Shan-shan Zhang, Zheng-Yi Tang

**Affiliations:** 1 Department of Endocrinology, Shanghai East Hospital, School of Medicine, Tongji University, Shanghai, China; 2 Department of Endocrinology, Zhongshan Hospital, Fudan University, Shanghai, China; 3 Department of Endocrine and Metabolic Diseases, Shanghai Institute of Endocrine and Metabolic Diseases, Ruijin Hospital, Shanghai Jiao Tong University School of Medicine, Shanghai, China; 4 Department of Endocrine and Metabolic Diseases, Shengli Oilfield Central Hospital, Shandong, China

**Keywords:** cardiovascular events, diabetic foot ulcers, mortality, serum uric acid, severity

## Abstract

**Background:**

Diabetic foot ulcers (DFU) as one of the most severe complications of diabetes was characterized by high mortality and incidence of cardiovascular events. Serum uric acid (UA) was well known for cause of gout which was closely related with cardiovascular complications, but also known as the main antioxidant in extracellular fluid. In this study, we aimed to investigate whether serum UA was related with severity and prognosis of patients with DFU.

**Design and methods:**

535 patients with diabetes and firstly diagnosed with DFU hospitalized in Ruijin hospital was consecutively recruited. Concentration of serum UA was examined when admission. Participants were grouped into three groups according to tertiles of their serum UA level. These patients were followed up for an average of 48 months to observe the outcomes, including wound healing, non-fatal cerebral and cardiovascular events (NCCE) and all-cause death. The association of serum UA concentration with the severity of disease evaluated through Wagner and Infection degree, and risk of outcomes were analyzed through partial correlation and Cox regression analysis, respectively.

**Results:**

In bivariate correlation analysis, UA was negatively correlated with the Wagner (r=-0.159, P <0.001) and Infection degree (r=-0.171, P <0.001). In partial correlation, UA was still negatively and independently correlated with the Wagner (r=-0.74, P <0.001) and Infection degree (r=-0.190, P <0.001) after controlling for age, sex, type of diabetes, duration of diabetes, duration of DFU, PAD, HbA1c, serum creatine and urine protein excretion in 24hours. In terms of Cox regression analysis, UA was positively and independently related with the risk of NCCE (HR = 1.002, 1.000 to 1.004, P = 0.011), all-cause mortality (HR = 1.003, 1.001 to 1.004, P = 0.006) and healing rate (HR = 1.001, 1.000 to 1.003,P = 0.042) after adjusting for age, sex, type of diabetes, duration of diabetes, duration of DFU, history of stroke, history of CAD, PAD, HbA1c, serum creatine and urine protein excretion in 24 hours.

**Conclusion:**

UA might exert a beneficial effect in the context of DFU wound repair, but was an important risk factor for NNCE and all-cause mortality in patients with DFU.

## 1 Introduction

Diabetic foot ulcers (DFU), as a disastrous complication of diabetes, was characterized by a high rate of disability and death (1, 2). Numerous studies revealed that cardiovascular and neuronal abnormality contributed to the development of DFU and its poor prognosis. In our previous studies, we explored potential predictors for adverse outcomes in DFU patients, including poor cardiac function (3), cardiac autonomic neuropathy (4), poor nutrition (5) and low estimated glomerular filtration rate (6). In recent years, UA was likely reported to be related with cardiovascular disease. However, the conclusion still remained controversial. It is interesting to analyze the association between UA and DFU severity and prognosis which was closely related with cardiovascular complications.

On the one hand, increased UA was well known to increase the development of gout. The published observational study reported that gout was associated with higher incidences of stroke, ischemic heart disease, heart failure and increased calcification of vessels (7). A retrospective cohort study from Taiwan (8)reported that patients with gout had higher risks of stroke than control group. On the other hand, clinical trial found that uric acid treatment reduced the infarct growth in women with acute ischemic stroke (9). Basic study also found that uric acid, acted as an antioxidant, could attenuate neuronal loss, cerebral perfusion impairment and neurological deficits in traumatic brain injury mice (10). Thus, the effect of uric acid on the prognosis of DFU patients still remain elusive, as DFU population was well known for a poor peripheral vascular and nervous condition.

In this observational prospective study, we assessed whether serum uric acid level was associated with severity and outcomes which included wound healing rate, non-fatal cerebral cardiovascular events (NCCE) incidence and all-cause mortality in DFU population. To the best of our knowledge, this was the only long-term real-world study of such scale to investigate these associations.

## 2 Methods

### 2.1 Subjects

A total of 535 patients hospitalized in our center who filled all inclusion criteria and none of exclusion criteria from March, 2009 to December 2013 were recruited. In brief, the inclusion criteria included: (1) aged≥18 years old when admitted; (2) the diagnosis of diabetes was confirmed or were in anti-diabetic medication or insulin therapy; (3) with at least one ulcer located at or below the ankle; (4) with a full record of serum uric acid concentration. The excluded criteria were: (1) not met all of the inclusion criteria; (2) on long-term glucocorticosteroid or immunosuppressive therapy; (3) having severe organic failure, Cushing’s disease, cancerous ulcers, or other fatal diseases; (4) having DFU occurrence before; (5) having major amputation before; (6) on dialysis treatment;(7) having kidney transplant before; (8) a lack of UA measurement.

### 2.2 Measurements and DFU severity classification

All recruited patients were interviewed when admitted to our department to obtain basic information, including age, sex, duration of diabetes, duration of DFU, occurrence conditions of DFU, previous and present therapy for diabetes, a past history of hypertention, cardiac disease, stroke and other chronic diseases, family history. A careful physical examination was performed by an experienced physician in our center in 24hours after admitted. The blood and urine samples were collected for a battery of biochemical tests after 10-hour fasting once admitted. The biochemical tests including serum concentration of UA, creatine, and BUN; parameters of liver functions; serum electronic minerals; fasting blood glucose; fasting blood lipid profiles; HbA1c; general tests of blood and urine; 24 h micro-albuminuria quantification.

Patients’ ulcers depth was graded according to Wagner’s Classification (11): grade 0 (pre- or postulcerative lesion), grade 1 (partial or full thickness ulcer), grade2 (probe to tendon or capsule), grade 3 (deep with osteomyelitis), grade 4 (partial foot gangrene), grade 5 (whole foot gangrene).

The infection degree of DFU patients were classified into four grade including uninfected, mild, moderate and severe, according to the Infectious Diseases Society of America’s diabetic foot infection classification system (12). Uninfected: without purulence or any manifestations of inflammation. Mild infection:≥ 2 manifestations of inflammation (purulence or erythema, pain, tenderness, warmth, or induration); any cellulitis or erythema extends <2 cm around ulcer, and infection is limited to skin or superficial subcutaneous tissues; no local complications or systemic illness. Moderate infection: infection in a patient who is systemically well and metabolically stable but has ≥1 of the following: cellulitis extending >2 cm; lymphangitis; spread beneath fascia; deep tissue abscess; gangrene; muscle, tendon, joint, or bone involvement. Severe infection: infection in a patient with systemic toxicity or metabolic instability (e.g. fever, chills, tachycardia, hypotension, confusion, vomiting, leukocytosis, acidosis, hyperglycemia, or azotemia).

Diabetic peripheral neuropathy (DPN) (13) was diagnosed when 2 of the following criteria were met: (1) neuropathic pain, anesthesia or other symptoms of paraesthesia; (2)abnormal pinprick sensation of the lower limbs or altered 10-g Semmes–Weinstein monofilament test; or (3) diminished ankle reflexes. Peripheral artery disease (PAD) (13)was diagnosed if 1 or more lower limb artery occlusions were spotted by Doppler ultrasound and/or ankle-brachial pressure index (ABI) < 0.9 in either of the limbs.

### 2.3 Outcomes and follow-up

Primary ulcers’ healing was defined as full coverage of skin or crusta on the primary ulcer without major amputation of the limb. All deaths and causes that occurred during the hospitalization and follow-up periods were recorded. Deaths caused by ischemic heart disease, acute congestive heart failure (AHF), cardiogenic shock, pulmonary embolism, or sudden death were defined as deaths of cardiac source. Nonfatal cerebral and cardiovascular events (NCCE) included nonfatal cerebrovascular events, nonfatal ischemic heart disease, nonfatal emergency-needed congestive heart failure, nonfatal severe ventricular arrhythmia, nonfatal cardiogenic shock and pulmonary embolism.

The follow-up was performed during clinic visits (twice or 3 times a week before ulcers healed; once in 1 or 3 months after ulcers healed) or their readmission time. For patients receiving therapy elsewhere, the follow-up was done through telephoning. All of the above events and their occurrence times were determined and recorded. All of the studied patients were followed until death or until June 1st, 2016. The data analysis was done from June,2023 to Decenmber,2023.

### 2.4 Statistical analysis

Quantitative variables were described by the mean ± SD or median (range) according to their distribution. T-test was used for normally distributed variables, otherwise, Mann-Whitney was used, so as to analyze differences between two groups. Discontinuous variables were expressed using frequency, and comparisons were made using the χ^2^ test. To test the association between UA and Wagner degree or infection degree, correlation analysis (2-tailed) was applied. In bivariate correlation analysis, spearman correlation was performed. Then, the following factors were added as covariates in partial correlation analysis step wisely: age, sex, type of diabetes, duration of diabetes, duration of DFU, PAD, HbA1c, serum creatinine, 24h urine protein excretion. To analyze the association between risk of outcomes and serum urine acid, hazard ratios with 95% CIs were first calculated using univariate Cox proportional-hazards models. Then, the following factors were included as confounders to ascertain hazard ratios for outcomes in multiple Cox regression models: age, sex, type of diabetes, duration of diabetes, duration of DFU, history of stroke, history of CAD, Wagner degree, infection degree, PAD, HbA1c, serum creatine, 24h urine protein excretion. In Cox regression analysis, the Backward LR model was applied. All the statistical analysis was performed using the SPSS statistical system (version 28.0.1.1; IBM corp.). A p value <.05 was considered statistically significant.

## 3 Results

By the end of the study, the median follow-up time was 38months, ranging from 0.13 to 84 months.

### 3.1 Baseline characteristics

The baseline characteristics of patients were stratified into three groups according to serum UA levels (tertile1: ≤229, tertile 2: 229 to 307, tertile3: >307). The demographic characteristics were listed in **Table 1**. At the beginning of this study, no statistical difference was observed in sex, type of diabetes, duration of diabetes, duration of DFU, body height, BMI, systolic blood pressure, diastolic blood pressure, low density lipoprotein cholesterin (LDL-C), alkaline phosphatase, total bilirubin, direct bilirubin, potassium, urine protein excretion in 24 hours, white and red blood cell count (all P>0.05) in DFU patients of different UA tertiles. Patients with higher tertile of urine acid were more likely to be older, with smaller ulcers of both lower Wagner and infection degree, lower C-reaction protein (CRP) concentration and also higher body weight. With respect to complications, more patients of tertile 1 tended to develop PAD than tertile 2 and 3, but no significant existed in DPN among patients of three tertiles. As for glucose control, both fasting blood glucose and HbA1c were lower, but fasting C peptide was higher in patients with higher UA. In terms of blood lipid profile, patients of lower UA tertile group were shown to have significantly lower triglyceride, total cholesterol (TC) and high-density lipoprotein cholesterol (HDL-C) concentration. Furthermore, patients of higher UA tertile group tended to have higher serum concentration of total protein, albumin, blood urine nitrogen, sodium, chlorine, phosphorus.

**Table 1:** Baseline characteristics of different serum uric acid tertiles.

Uric acid	Tertile 1	Tertile 2	Tertile 3	Total	P-value
	≤229 umol/L	229~307 umol/L	>307 umol/L	59~684 umol/L	
	N=183	N=176	N=176	N=535	
Age (years old)	64.76 ± 12.60	66.68 ± 11.50	67.98 ± 12.09	66.45 ± 12.13	0.04*
Sex (male/total)	113/183	115/176	123/176	351/535	0.267
Type (T2DM/total)	180/183	176/176	175/176	531/535	0.186
Duration of DM (months)	10.0 (0.01,40)	10.0 (0.02,180)	10.0 (0.01,42)	10.0 (0.01,180)	0.808
Duration of DFU (days)	30 (1,1800)	30 (2,2000)	50 (1,2500)	30 (1,2500)	0.179
Ulcer Area (cm^2^)	12 (0.75,269)	10 (0.25,210)	9 (0.25,200)	10 (0.25,269)	0.04*
Wagner degree					
0	0	1	0	1	<0.001**
1	20	27	39	86	
2	38	50	33	121	
3	39	40	49	128	
4	69	50	49	168	
5	17	8	6	31	
Infection degree					
0	6	15	15	36	<0.001**
1	37	39	41	117	
2	66	74	79	219	
3	74	48	41	163	
DPN (with/total)	45/183	41/176	51/176	137/535	0.4399
PAD (with/total)	81/183	79/176	103/176	263/535	<0.001**
Weight (kg)	65.14 ± 18.34	64.89 ± 11.94	69.52 ± 14.43	66.43 ± 15.11	0.027*
Height (cm)	166.80 ± 13.18	166.26 ± 12.20	165.42 ± 20.74	166.18 ± 15.57	0.810
BMI (kg/m^2^)	22.85 ± 4.45	23.17 ± 3.63	23.89 ± 4.06	23.28 ± 4.05	0.190
SBP (mmHg)	134.64 ± 2.00	139.60 ± 20.85	137.70 ± 19.62	137.27 ± 20.57	0.072
DBP (mmHg)	76.69 ± 10.78	77.66 ± 13.22	76.71 ± 12.81	76.81 ± 12.29	0.482
HbA1c (%)	9.47 ± 2.46	9.04 ± 2.23	8.59 ± 2.25	9.05 ± 2.34	0.002**
FBG (mmol/L)	9.28 ± 4.51	8.07 ± 3.78	7.62 ± 3.75	8.34 ± 4.09	<0.001**
Fasting C peptide (ng/ml)	0.66 (0.03,7.54)	1.02 (0.003,5.12)	1.23 (0.107,9.00)	(0.003,9.00)	<0.001**
2h C peptide (ng/ml)	1.54 (0.05,102.4)	2.73 (0.003,10000)	2.81 (0.234,339.4)	(0.003,1000)	<0.001**
TC (mmol/L)	3.61 ± 1.00	3.91 ± 0.96	3.90 ± 1.09	3.80 ± 1.03	0.007**
TG (mmol/L)	1.03 (0.36,5.16)	1.04 (0.30,9.13)	1.195 (0.42,11.90)	1.09 (0.30,11.90)	<0.001**
HDL-C (mmol/L)	0.92 ± 0.34	1.02 ± 0.36	0.94 ± 0.33	0.96 ± 0.35	0.020*
LDL-C (mmol/L)	2.33 ± 0.85	2.49 ± 1.12	2.36 ± 0.93	2.39 ± 0.97	0.255
ALT (U/L)	16 (3,98)	16 (2,214)	16 (13,171)	16 (2,214)	0.961
AST (U/L)	16 (4,366)	17 (4,129)	17 (5,127)	17 (4,366)	0.468
ALP (U/L)	79 (26,772)	77 (14,367)	74 (36,622)	76 (14,772)	0.147
TBil (umol/L)	7.5 (1.8,38.5)	8.1 (1.9,26.8)	8.5 (0.6,33.4)	8.1 (0.6,38.5)	0.189
DBil (umol/L)	2.55 (0.4,55)	2.5 (0.4,12.3)	2.56 (0.4,16.9)	2.51 (0.4,55)	0.848
Total protein (g/L)	61.99 ± 7.03	63.74 ± 6.51	64.21 ± 6.25	63.30 ± 6.67	0.004**
Albumin (g/L)	31.55 ± 5.17	33.77 ± 5.44	34.37 ± 5.46	33.21 ± 5.49	<0.001**
BUN (mmol/L)	5.60 ± 6.25	6.32 ± 2.63	9.91 ± 9.96	7.25 ± 7.18	<0.001**
Creatine (mmol/L)	63.99 ± 47.33	77.87 ± 71.44	139.55 ± 178.65	93.44 ± 118.22	<0.001**
Uric acid (umol/L)	186.26 ± 35.92	266.89 ± 22.77	404.36 ± 85.16	284.53 ± 105.5	<0.001**
K (mmol/L)	3.95 ± 0.47	4.02 ± 0.50	4.05 ± 0.47	4.01 ± 0.47	0.148
Na (mmol/L)	135.74 ± 5.60	137.57 ± 3.98	137.79 ± 4.58	137.02 ± 4.86	<0.001**
Cl (mmol/L)	99.80 ± 3.86	101.25 ± 4.37	101.95 ± 4.03	100.98 ± 4.32	<0.001**
Ca (mmol/L)	2.06 ± 0.23	2.11 ± 0.19	2.13 ± 0.21	2.10 ± 0.21	0.014*
P (mmol/L)	1.09 (0.31,20.36)	1.16 (0.52,2.91)	1.25 (0.47,1.86)	1.17 (0.31,20.36)	<0.001**
Urine protein excretion (mg/24h)	0.324 (0.02,300)	0.2285 (0.1,8.06)	0.270 (0.044,82)	0.270 (0.01,300)	0.155
WBC (10^9/L)	9.05 ± 4.42	8.32 ± 3.79	9.07 ± 5.18	8.82 ± 4.51	0.206
RBC (10^9/L)	3.65 ± 0.65	3.74 ± 0.85	3.76 ± 0.69	3.72 ± 0.73	0.329
Hb (g/L)	106.29 ± 21.78	109.97 ± 21.54	111.55 ± 23.29	109.25 ± 22.28	0.075
PLT (10^9/L)	240.99 ± 104.26	224.69 ± 93.02	212.45 ± 84.61	225.99 ± 94.82	0.023*
CRP (mg/L)	35.60 (0.16,334)	9.12 (0.16,250)	11.30 (0.15,355)	17.55 (0.15,355)	<0.001**

*P<0.05; **, P<0.01; T2DM, type 2 diabetes mellitus; DFU, diabetic foot ulcers; DPN, diabetic peripheral neuropathy; PAD, peripheral arterial disease; BMI, body mass index; SBP, systolic blood pressure; DBP, diastolic blood pressure; HbA1c, glycated haemoglobin; FBG, fasting blood glucose; TC, total cholesterol; TG, triglyceride; HDL-C, high density lipoprotein cholesterol; LDL-C, low density lipoprotein cholesterol; ALT, alanine aminotransferase; AST, Aspartate aminotransferase; ALP, alkaline phosphatase; TBil, total bilirubin; DBil, Direct Bilirubin; BUN, blood urea nitrogen; K, potassium; Na, sodium; Cl, chlorin; Ca, calcium; P, phosphorus; WBC, white blood cell; RBC, red blood cell; Hb, haemoglobin; PLT, blood plate; CRP, C-reactive protein.

### 3.2 Association between UA concentration and severity of DFU

As shown in **Table 2**, In bivariate correlation analysis, UA concentration was significantly and negatively correlated with both Wagner degree (r=-0.159, P<0.001) and infection degree (r=-0.171, P<0.001). After adjusted for age, sex, type of diabetes, PAD, duration of diabetes and duration of DFU, UA concentration was still significantly and negatively associated with both Wagner degree (r=-0.131, P = 0.003) and infection degree (r=-0.126, P = 0.004). In addition, when HbA1c, serum creatine, 24h urine protein excretion further entered the model, the association of UA concentration with Wagner degree (r=-0.74, P<0.001) or infection degree (r=-0.190, P<0.001) were more obvious and still significant.

**Table 2:** Association between serum urine acid and Wagner or infection degree.

Model	Wagner degree		Infection degree	
	r	P	r	P
Model 1	-0.159	<0.001**	-0.171	<0.001**
Model 2	-0.131	0.003*	-0.126	0.004*
Model 3	-0.74	<0.001**	-0.190	<0.001**

Model 1: Bivariate partial correlation analysis;.

Model 2: controlling for age, sex, type of diabetes, PAD, duration of diabetes and duration of DFU.

Model 3: controlling for variates in model 2 and HbA1c, serum creatine, 24h urine protein excretion;

Abbreviations were listed in **Table 1**.

### 3.3 Healing rate

Of the 535 DFU patients, 365 (68.22%) patients’ ulcers attained primary healing before died or by the end of follow-up period. The cumulating healing rate in 3, 6, 12 months of the whole cohort was 51%, 60% and 67%.

For patients of the lower and upper UA tertile group, the cumulating healing rate in 3,6,12 months was 50% and 49%, 59% and 57%, 68% and 64%, respectively. In univariate COX regression analysis, UA was not significantly related with healing rate (HR = 1.000, 0.999 to 1.001, P = 0.654). After adjusting for age, sex, type of diabetes, duration of diabetes, duration of DFU, history of stroke, history of CAD, Wagner degree, infection degree and PAD, UA concentration was still not significantly associated with healing rate of DFU patients (HR = 1.000, 0.999 to 1.001, P = 0.817) (**Table 3**). However, when HbA1c, serum creatine and 24h urine protein excretion entered the above model, UA concentration was shown to be positively and significantly related with healing rate of DFU patients (HR = 1.001, 1.000 to 1.003, P = 0.042) (**Table 3**).

**Table 3:** HRs of the association between UA and DFU Outcomes in Univariate and Multivariate Cox Proportional-hazard Model.

Outcomes	HR	95%CI	P-value	Covariates in model
Healing				
Model 1	1.000	0.999 to 1.001	0.654	–
Model 2	1.000	0.999 to 1.001	0.817	History of CAD
Model 3	1.001	1.000 to 1.003	0.042*	Serum creatine, HbA1c, Infection degree
NCCE				
Model 1	1.003	1.001 to 1.004	0.004*	–
Model 2	1.002	1.000 to 1.004	0.016*	Age, history of stroke, history of CAD
Model 3	1.002	1.001 to 1.004	0.011*	Age, history of CAD
Death				
Model 1	1.002	1.001 to 1.004	0.004*	–
Model 2	1.002	1.000 to 1.003	0.014*	Age, PAD, Wagner degree
Model 3	1.003	1.001 to 1.004	0.006*	Age, infection degree, HbA1c

Model 1, univariate analysis.

Model 2, adjusted for age, sex, type of diabetes, duration of diabetes, duration of DFU, history of stroke, history of CAD, Wagner degree, infection degree, PAD.

Model 3, adjusted for variates in model 2 and HbA1c, serum creatine, 24h urine protein excretion.

CAD, coronary heart disease; other abbreviations were listed in **Table 1**.

### 3.4 Incidence of non-fatal cerebral cardiovascular event

By the end of the whole follow-up period, 190 of 535 (19%) DFU patients experienced NCCE. The cumulating incidence of NCCE in 3,6,12 months was 6%,8% and 9%.

The cumulating NCCE incidence of lower and upper UA tertile group in 3,6,12 months was 2% and 9%, 4% and 12%, 4% and 15%, respectively. UA concentration and NCCE incidence was tested to be positively and significantly associated in univariate COX regression (HR = 1.003, 1.001 to 1.004, P = 0.004) (**Table 3**). In addition, the positive association between UA concentration and NCCE incidence in DFU patients was not affected by the adjustment of age, sex, type of diabetes, duration of diabetes, duration of DFU, history of stroke, history of CAD, Wagner degree, infection degree and PAD (HR = 1.002, 1.000 to 1.004, P = 0.016) (**Table 3**). When HbA1c, serum creatine and 24h urine protein excretion was further adjusted, UA concentration was still independently and significantly associated with NCCE incidence (HR = 1.002, 1.001 to 1.004, P = 0.011) (**Table 3**).

### 3.5 All-cause mortality

Of the whole cohort, 135 (25.2%) DFU patients died during follow up. The cumulating all-cause mortality in 3,6,12 months was 5%,8% and 11%.

The cumulating all-cause mortality of lower and upper UA tertile group in 3,6,12 months was 3% and 8%, 6% and 10%, 11% and 14%, respectively. The slightly but significantly positive association between all-cause mortality and UA concentration of DFU patients was observed in univariate Cox regression analysis (HR = 1.002,1.001 to 1.004, P = 0.004) (**Table 3**). After further adjusted for age, sex, type of diabetes, duration of diabetes, duration of DFU, history of stroke, history of CAD, Wagner degree, infection degree and PAD, UA concentration was still positively and significantly related with all-cause mortality (HR = 1.002,1.001 to 1.003, P = 0.014) (**Table 3**). Finally, UA concentration was demonstrated to be positively and independently associated with all-cause mortality in DFU patients (HR = 1.003, 1.001 to 1.004, P = 0.006) after HbA1c, serum creatine and 24h urine protein excretion were further adjusted (**Table 3**).

## 4 Discussion

In this study, we firstly analyzed the association between UA concentration and severity of DFU patients which was reflected through Wagner and infection degree. We found that circulating UA concentration was negatively and independently correlated with both Wagner and infection degree. Then, the relationship of UA concentration with outcomes in DFU patients were analyzed which included healing rate, NCCE incidence and all-cause mortality. We surprisingly found that UA concentration was an independently protective factor of healing rate, but an independently risk factor of NCCE incidence and all-cause death mortality in DFU population. To the best of our knowledge, this is the first study investigating the relationship between circulating UA on disease severity and the predicting role of serum UA for prognosis of DFU patients.

First of all, we found the significantly and independently negative correlation of circulating UA concentration with both Wagner and infection degree. From the perspective of clinical study, we speculated that the hypouricaemia was caused by an increased fractional excretion of UA at renal tubule resulting from the osmotic diuretic effect of glucose (14) in patients of higher Wagner or infection degrees where a poorer control of blood glucose was always observed. However, this speculation was broken, as the negative relation between UA and Wagner or infection degrees was still significant when HbA1c was adjusted. This means that the negative correlation of UA concentration with severity of DFU was independent of blood glucose control. In fact, it had been reported in our previously published study that Wagner degree was positively correlated with the incidence of both heart failure (3) and malnutrition (5) in DFU patients. Gastrointestinal tract, as one of the main organs that providing substrates for UA production, was always structurally edematous and functionally impaired in DFU patients of higher Wagner degrees. Thus, the reduced provision of UA substrates in DFU patients of higher disease severity might be one of possible reasons. From the perspective of basic research, UA had been reported to be the main antioxidant in tissue fluid very early, accounting for almost two thirds antioxidant activity of antioxidants (15). UA was shown to protect the normal function of endothelium from oxidative stress in DFU progression (16), resulting in a lower Wagner degree. This could also explain the negative relation between UA and infection degree, since infection caused the essential source of reactive oxygen species (ROS). However, there reported a completely different result in other study (17) which found a positive relationship between UA concentration in wound fluid and severity of DFU wound. This result seemed to be contradictive with our study. In fact, only wounds without infection were included in the aforementioned research, which was different from our study. Most importantly, it is UA concentration in wound fluid not in circulation that was studied in this study. Thus, the result from our study was not in conflict with the above. In addition, because infection was one of the most important factors for poor prognosis of DFU and measuring UA in blood is more accurate and convenient than in wound fluid. Result from our study was more persuasive and applicable than the above-mentioned research.

Secondly, for the very first time, we found the positive relationship between circulating UA and healing rate of DFU patients. No clinical reference about the relation between serum UA concentration and wound healing were published before. For basic studies, UA was recently reported to upregulate the expression of stem cell factor (SCF) and basic fibroblast growth factor (bFGF) in keratinocytes (18). On the one hand, bFGF was one of the critical components accelerating angiogenesis and tissue regeneration by promoting the migration of dermal fibroblasts and endothelial cells associated with matrix formation and remodeling in wound healing process (19). Previously, application of bFGF was demonstrated to accelerate wound healing while achieving a highly organized extracellular matrix and enhanced angiogenesis *in vivo (*20). On the other hand, SCF was also shown to improve wound closure in diabetic mice through increasing HIF-1α and vascular endothelial growth factor (VEGF) levels in these wounds (21). Therefore, the significantly positive relation between UA and wound healing rate might be explained by the upregulation of SCF and bFGF derived from keratinocytes by UA where further basic experiments are needed.

Thirdly, the positive correlation of UA concentration with NCCE incidence and all-cause mortality of DFU was observed in our study. Prospective cohort studies of general population have indicated the U-shaped relationship between serum UA concentrations and mortality outcomes (22, 23), hinting at the adverse effect of low and high UA levels. However, the appropriate target for UA levels among patients with diabetes is inconclusive. Previous studies conducted in patients with type 2 diabetes showed that high UA level was associated with the augmented risks of all-cause and CVD mortality. In recently published study, Benchao Li (24) indicated that higher serum UA levels were associated with increased risks of all-cause and CVD mortality in diabetes through the analysis of 7,101 patients with diabetes from NHANES 1999–2018 and a meta-analysis. Ioachimescu (25) also found that the 1 mg/dL increment in UA level was associated with 21% higher risk of all-cause mortality in a retrospective cohort study of 535 patients with diabetes. Kleber (26) reported causal HRs corresponding to each 1-mg/dl increase in genetically predicted uric acid concentration were significant for 77% increase in cardiovascular death and 141% increase in sudden cardiac death in a mendelian randomization study.

Last but not least, several potential limitations should be noticed in our study. For one thing, a single baseline circulating UA concentration was used to predict outcomes. Our study, as an observational and prospective real-world study, did not aim to assess the relationship between the change of UA and prognosis. A single measurement of UA level to predict outcomes was a simplified and practical approach, similar to what was done in previously reported studies (22–25). In the next place, details on diuretics and urate‐lowering agents were lacking in our study. It was indicated that the association between UA and mortality is independent of diuretic use in the first National Health and Nutrition Examination Survey (27), and we believed that diuretic use may not affect our results. Moreover, both ischemic and hemorrhagic strokes were taken as NCCE in our study. It remained unknown if the role of UA played in these two conditions was different or not. More basic and clinical investigations were needed to address the problem above mentioned.

## 5 Conclusion

The real-world prospective study with a 4-year follow-up of Chinese DFU patients revealed the negative relation of serum UA concentration with wound severity and a significant positive correlation with wound healing rate after adjusting for relevant rates. But higher circulating UA concentration predicted higher incidence of NCCE and all-cause mortality in DFU populations. Clinicians should not ignore the control of UA concentration in consideration of cardiovascular condition and survival longevity.

## Data Availability

The data that support the findings of this study are available from the corresponding author upon reasonable request. The data are not publicly available due to restrictions containing information that could compromise the privacy of research participants.
